# Advanced Non-Destructive Testing Simulation and Modeling Approaches for Fiber-Reinforced Polymer Pipes: A Review

**DOI:** 10.3390/ma18112466

**Published:** 2025-05-24

**Authors:** Jan Lean Tai, Mohamed Thariq Hameed Sultan, Andrzej Łukaszewicz, Jerzy Józwik, Zbigniew Oksiuta, Farah Syazwani Shahar

**Affiliations:** 1 Department of Aerospace Engineering, Faculty of Engineering, Universiti Putra Malaysia, Serdang 43400, Selangor, Malaysia; gs63398@student.upm.edu.my (J.L.T.); farahsyazwani@upm.edu.my (F.S.S.); 2 Laboratory of Biocomposite Technology, Institute of Tropical Forest and Forest Product (INTROP), Universiti Putra Malaysia, Serdang 43400, Selangor, Malaysia; 3 Aerospace Malaysia Innovation Centre [944751-A], Prime Minister’s Department, MIGHT Partnership Hub, Jalan Impact, Cyberjaya 63600, Selangor, Malaysia; 4 Institute of Mechanical Engineering, Faculty of Mechanical Engineering, Bialystok University of Technology, Wiejska St. 45C, 15-351 Bialystok, Poland; 5 Department of Production Engineering, Faculty of Mechanical Engineering, Lublin University of Technology, Nadbystrzycka St. 36, 20-618 Lublin, Poland; j.jozwik@pollub.pl; 6 Institute of Technical Sciences and Aviation, The University College of Applied Sciences in Chelm, Pocztowa St. 54, 22-100 Chełm, Poland; 7 Institute of Biomedical Engineering, Faculty of Mechanical Engineering, Bialystok University of Technology, Wiejska St. 45C, 15-351 Bialystok, Poland; z.oksiuta@pb.edu.pl

**Keywords:** fiber-reinforced polymer (FRP), carbon fiber-reinforced polymer (CFRP), non-destructive testing (NDT), simulation, modeling, finite element method (FEM)

## Abstract

Fiber-reinforced polymer (FRP) pipes have emerged as a preferred alternative to conventional metallic piping systems in various industries, including chemical processing, marine, and oil and gas industries, owing to their superior corrosion resistance, high strength-to-weight ratio, and extended service life. However, ensuring the long-term reliability and structural integrity of FRP pipes presents significant challenges, primarily because of their anisotropic and heterogeneous nature, which complicates defect detection and characterization. Traditional non-destructive testing (NDT) methods, which are widely applied, often fail to address these complexities, necessitating the adoption of advanced digital techniques. This review systematically examines recent advancements in digital NDT approaches with a particular focus on their application to composite materials. Drawing from 140 peer-reviewed articles published between 2016 and 2024, this review highlights the role of numerical modeling, simulation, machine learning (ML), and deep learning (DL) in enhancing defect detection sensitivity, automating data interpretation, and supporting predictive maintenance strategies. Numerical techniques, such as the finite element method (FEM) and Monte Carlo simulations, have been shown to improve inspection reliability through virtual defect modeling and parameter optimization. Meanwhile, ML and DL algorithms demonstrate transformative capabilities in automating defect classification, segmentation, and severity assessment, significantly reducing the inspection time and human dependency. Despite these promising developments, this review identifies a critical gap in the field: the limited translation of advanced digital methods into field-deployable solutions specifically tailored for FRP piping systems. The unique structural complexities and operational demands of FRP pipes require dedicated research for the development of validated digital models, application-specific datasets, and industry-aligned evaluation protocols. This review provides strategic insights and future research directions aimed at bridging the gap and promoting the integration of digital NDT technologies into real-world FRP pipe inspection and lifecycle management frameworks.

## 1. Introduction

The increasing demand for advanced materials with superior mechanical, chemical, and thermal performances has driven the widespread adoption of fiber-reinforced polymer (FRP) composites across industries such as aerospace, automotive, marine, civil infrastructure, oil and gas, and chemical processing. Among the various applications, FRP pipes have emerged as a compelling alternative to conventional metallic piping systems owing to their exceptional corrosion resistance, lightweight nature, ease of installation, and reduced lifecycle costs [1]. These attributes make FRP piping systems particularly attractive for harsh operating environments, where traditional metals are prone to corrosion, scaling, and fatigue-related failures [2,3,4,5,6,7].

Despite these advantages, FRP pipes are not immune to the defects that can arise during manufacturing, installation, and in-service operations. Such defects, including voids, delamination, fiber misalignment, and geometric deformations, can significantly compromise structural integrity and long-term reliability if left undetected [8,9]. Therefore, the implementation of robust non-destructive testing (NDT) strategies is essential to ensure quality assurance, structural health monitoring, and life-cycle management of FRP piping assets [10].

Conventional NDT methods, such as ultrasonic testing (UT), radiographic testing (RT), acoustic emission testing (AET), infrared thermography (IRT), and visual inspection (VI), have been extensively applied in various industries. However, these techniques face considerable limitations when applied to FRP materials, primarily because of their anisotropic, heterogeneous, and multilayered nature [11,12]. Complex failure modes such as delamination, fiber breakage, and matrix cracking are often difficult to detect using traditional NDT approaches, which typically rely on surface or near-surface interrogation and operator expertise. Additionally, the low density and poor contrast in radiographic imaging, limited depth resolution in thermography, and variability in human interpretation further constrain the reliability and efficiency of these conventional methods for FRP inspection [13].

Recognizing these challenges, recent research and industrial practices have increasingly turned to advanced NDT approaches that leverage digital technologies, including numerical modeling, simulation, machine learning (ML), deep learning (DL), and digital twin frameworks. These technologies offer transformative potential to enhance defect detection sensitivity, automate data interpretation, and support predictive maintenance through real-time monitoring. The finite element method (FEM), boundary element method (BEM), and Monte Carlo simulations have demonstrated significant capability in simulating defect behaviors, optimizing inspection parameters, and improving the model-assisted probability of detection (MAPOD). ML and DL algorithms have enabled the automated classification, segmentation, and severity assessment of defects across various NDT modalities [14].

Although substantial progress has been made in applying these digital techniques to general composite materials, their application to FRP piping systems, characterized by large diameters, complex geometries, and multilayered structures, remains underexplored. This highlights the urgent need for research that specifically addresses the unique challenges associated with FRP pipes, including the development of validated digital models, application-specific datasets, and standardized evaluation protocols.

This review aims to bridge this knowledge gap by systematically examining the recent advancements in digital NDT methods tailored to composite materials. Drawing on 140 peer-reviewed studies published between 2016 and 2025, this study categorized and evaluated digital NDT strategies, including numerical simulations, ML, DL, and their integration into real-time monitoring systems. The insights provided herein offer a comprehensive reference for researchers and practitioners seeking to enhance the reliability, efficiency, and cost-effectiveness of FRP pipe inspection and lifecycle management using advanced digital technologies.

## 2. Overview of Non-Destructive Testing (NDT)

NDT is a cornerstone of quality assurance, structural health monitoring, and life cycle management in industries such as aerospace, oil and gas, civil infrastructure, maritime, and manufacturing. NDT is defined as the process of inspecting, testing, or evaluating materials, components, or assemblies for discontinuities, defects, or differences in characteristics without permanently altering their functionality or serviceability [15,16,17]. Unlike destructive testing methods that require cutting, breaking, or damaging the test material, NDT enables the in situ assessment of assets throughout their service life.

### 2.1. Conventional NDT Techniques and Their Applications

NDT methods have been widely applied to detect surface and subsurface discontinuities in metallic structures, welds, pipelines, pressure vessels, and structural components [15]. Common conventional NDT methods include the following.

- Ultrasonic testing (UT): High-frequency sound waves are transmitted to a material to detect internal flaws, characterize thickness, or evaluate bonding quality [18,19,20,21]. UT is effective for metallic and some composite materials, but may face limitations in highly attenuative or anisotropic materials such as FRP [22].
- Radiographic testing (RT): X-rays or gamma rays are employed to capture volumetric images of internal features. While highly effective for metallic welds and castings, RT struggles with low-density materials and requires radiation safety precautions [23,24,25,26].
- Penetrant testing (PT): This involves applying a dye or fluorescent liquid to the surface of a non-porous material to reveal surface-breaking cracks or porosity after a developer is applied [27,28,29]. PT is limited to open-to-surface defect detection.
- Magnetic particle testing (MT): Detects surface and near-surface discontinuities in ferromagnetic materials by applying magnetic fields and ferrous particles. MT is inapplicable to nonmagnetic materials, such as FRP.
- Acoustic emission testing (AET): Transient elastic waves generated by active damage events, such as crack growth or fiber fracture under stress [30,31,32,33,34,35]. AET is suitable for real-time monitoring, but is sensitive to background noise and requires expert interpretation [36,37,38,39].
- Infrared thermography (IRT): Thermal imaging is used to detect variations in surface temperature, which may indicate subsurface defects or delamination [40,41,42,43]. Effective for near-surface defects, but limited by depth resolution.
- Visual inspection (VI): The simplest form of NDT that relies on direct observation to detect visible defects. However, it is inherently limited to surface-level defects and is highly subjective.

### 2.2. Challenges of Conventional NDT on Composite Structures

Although these methods are well-established for metallic materials, their application in FRP composites, particularly FRP piping systems, presents significant challenges.

- Anisotropic and heterogeneous nature: FRP materials consist of fiber and resin matrices with directional properties, making wave propagation unpredictable in UT and other wave-based methods.
- Complex defect modes: FRP structures exhibit unique failure mechanisms, such as delamination, fiber breakage, matrix cracking, and void formation, many of which are difficult to detect using surface-focused methods such as PT or VI.
- Low density and low contrast: RT struggles to differentiate between the matrix and fiber in FRP materials owing to the low X-ray absorption contrast compared to metallic materials.
- Human interpretation dependency: Many NDT results, especially from AET and UT, rely heavily on the expertise of the inspector, leading to inconsistencies and subjectivity in defect characterization [44].
- Limited depth resolution: Techniques such as IRT and PT are constrained to detect surface or near-surface anomalies, which may not capture the critical subsurface damage in thick-walled FRP structures.
- Inspection time and cost: Traditional methods may require multiple setups, manual scanning, and subjective interpretation, leading to increased inspection times and costs, particularly for large or complex FRP installations.

### 2.3. The Need for Advanced NDT Approaches

To overcome these limitations, the industry is shifting toward advanced NDT, which integrates digital technologies, computational modeling, and data-driven algorithms to enhance defect-detection sensitivity, automate data interpretation, and improve inspection reliability. This includes the following:

- Numerical simulation and modeling (e.g., FEM and BEM): this allows for the prediction and visualization of defect interactions with inspection signals before physical testing [45,46,47,48].
- ML and DL: automated feature extraction, defect classification, and severity assessment using large datasets of inspection signals or images [49,50,51,52,53].
- Digital twin and real-time monitoring: this enables continuous asset health monitoring through virtual replicas of physical systems, integrating sensor data for predictive maintenance [54,55,56,57].
- MAPOD: quantifying inspection reliability through combined experimental and simulation-based probability of detection (POD) studies, reducing dependence on costly physical trials [58,59,60].
- Advanced imaging and sensing technologies: such as X-ray computed tomography (XCT) [61,62,63,64], phased array ultrasonic testing (PAUT) [65,66,67,68,69], eddy current testing (ECT) [70,71,72], high-frequency eddy current testing (HF-ECT) [73,74,75], and thermographic methods optimized through simulation [76,77,78].

These approaches offer significant benefits, including enhanced defect sensitivity and resolution, for detecting microdefects or complex damage modes. They also improve automation and consistency in defect evaluation, effectively reducing human bias and variability. Additionally, the optimized inspection parameters derived from the simulation contribute to reducing both inspection time and associated costs. Finally, these methods facilitate integration with predictive maintenance frameworks, condition-based monitoring (CBM), and comprehensive lifecycle management [79].

## 3. Fiber-Reinforced Polymer/Plastic

FRP is a composite material composed primarily of fibers and resins. This versatile material has numerous synonymous and dissimilar appellations that can cause perplexity among individuals. FRPs are manufactured by embedding fibers into a polymer matrix. This material surpasses traditional materials in several applications. To provide a more comprehensive understanding of FRPs, the following section provides an overview of the various available types of FRP.

### 3.1. Types of Fiber-Reinforced Polymer

FRP materials are a versatile group of composites specifically designed for particular applications and engineering requirements. The selection of the reinforcement fibers and polymer matrix plays a critical role in determining the characteristics of the material. The following are some fundamental types of FRP, and Table 1 provides a detailed overview.

1. Glass fiber-reinforced polymer (GFRP): GFRP composites utilize glass fibers as reinforcement. These materials are used in applications requiring high strength, corrosion resistance, and electrical insulation and are prevalent in the construction, automotive, and marine industries [80,81,82].
2. Carbon fiber-reinforced polymer (CFRP): CFRP composites employ carbon fibers as reinforcements with a noteworthy strength-to-weight ratio and stiffness. Apart from typical carbon material attributes such as high-temperature resistance, friction resistance, and corrosion resistance, CFRP possesses exceptional specific strength, specific modulus, and fatigue resistance [83,84]. CFRPs can be used as CFRP materials in aerospace, automotive, sporting goods, and structural engineering applications [85].
3. Aramid fiber-reinforced polymer (AFRP): AFRP composites incorporate aramid fibers, such as Kevlar, for reinforcement. These fibers impart high tensile strength, impact resistance, and flame resistance to the composite materials [86]. AFRP is commonly used in body armor, protective gears, and structural applications where high strength and durability are critical [87,88].
4. Basalt fiber-reinforced polymer (BFRP): BFRP composites that use basalt fibers for reinforcement are highly effective for high-temperature applications and are commonly used in the repair and reinforcement of infrastructure in the construction industry [89,90,91,92].
5. Natural fiber-reinforced polymer (NFRP): NFRP composites incorporate natural fibers, such as jute, hemp, or flax, as reinforcements and are valued for their environmental friendliness, nonhomogeneous composition, and diverse applications in automotive interiors, furniture, and construction [93,94,95]. Natural fibers have gained significant attention in recent years owing to their numerous advantages including cost-effectiveness, low density, exceptional flexibility, recyclability, and sustainability. However, their widespread use is limited by their relatively low impact strength and hydrophilicity [96].
6. Hybrid fiber-reinforced polymer (HFRP): HFRP composites are formulated by combining various fiber types, including natural/synthetic hybrid fibers, to achieve a comprehensive balance of properties such as strength, stiffness, and cost-effectiveness [97,98,99]. These composites were designed to satisfy the specific requirements of applications that require a combination of attributes [100,101,102,103].

**Figure 1:**
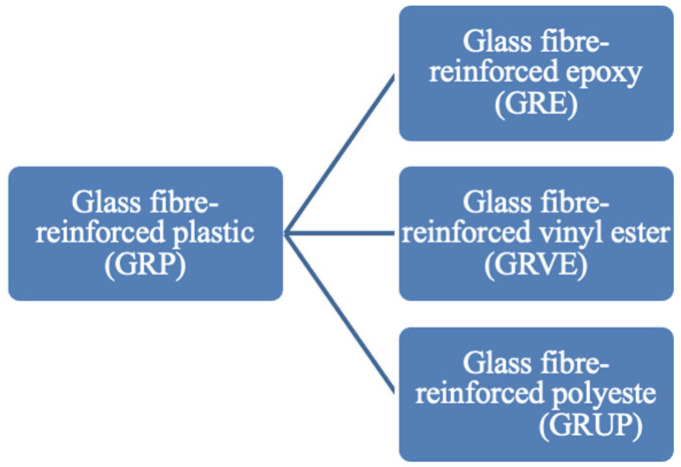
Various types of glass-fiber reinforced plastic.

CFRP and GFRP are widely used in the industry owing to their respective advantages, and GFRP is also referred to as GRP. Additionally, reinforced thermosetting resin plastic (RTR) has been used by some researchers because of the use of thermosetting resins. The term “resin” refers to a type of thermosetting plastic used to reinforce fiberglass. As shown in Figure 1, three primary types of thermosetting resins are used in the production of GRP: polyester, epoxy, and vinyl ester [104]. The use of different resins results in the production of three distinct GRP sub-types: glass fiber-reinforced epoxy (GRE) [105,106,107], glass fiber-reinforced vinyl ester (GRVE) [108,109,110,111], and glass fiber-reinforced polyester (GRUP) [112]. Table 2 summarizes the three main types of resin used in FRP manufacturing, highlighting their advantages, disadvantages, and typical mechanical properties for easier comparison [113,114,115].

#### Thermoplastic Resins in FRP Manufacturing

Although thermosetting resins, such as polyester, vinyl ester, and epoxy, have long been dominant in FRP manufacturing because of their excellent mechanical and thermal properties after curing, thermoplastic resins are gaining increasing attention as viable alternatives [116,117]. This shift is particularly notable in applications requiring recyclability, improved impact resistance, and thermoformability [118]. Unlike thermosets, thermoplastic resins do not undergo irreversible chemical crosslinking during processing. Instead, they can be softened and reshaped by heating, making them suitable for fusion bonding, recycling, and thermoforming processes. This property significantly enhances processing flexibility and end-of-life sustainability.

The common thermoplastic resins used in FRP applications include polypropylene (PP) [119,120,121,122,123], polyethylene terephthalate (PET) [124,125,126,127,128], polyether-ether-ketone (PEEK) [129,130,131,132], and polyamide (PA or Nylon) [133,134,135]. These resins offer several advantages, such as high impact resistance, good chemical and moisture resistance, thermal reprocessability, and recyclability. However, they also have limitations, including higher processing temperatures, equipment costs, and lower thermal stability than high-performance thermosets.

In the context of FRP pipe manufacturing, thermoplastic matrix composites have been explored for various applications, including chemical processing pipelines, oil and gas transportation lines, and water and wastewater infrastructure [136]. Emerging manufacturing methods, such as thermoplastic filament winding and fusion bonding, further enhance the industrial viability of thermoplastic-based FRP pipes. These methods enable the production of lightweight, corrosion-resistant, and recyclable piping systems that are suitable for a wide range of industrial applications. Table 3 provides a comparative summary of commonly used thermoplastic resins, highlighting their key properties, advantages, limitations, and typical applications to assist in the material selection for specific FRP manufacturing requirements.

### 3.2. Manufacturing Method

Understanding the various FRP classifications is crucial, as it paves the way for in-depth knowledge of the manufacturing processes involved in the production of FRP materials. The manufacturing of FRP materials encompasses a range of manufacturing processes, each of which is tailored to a specific product type and characteristics. The four commonly employed manufacturing methods are as follows.

1. Pultrusion process: Pultruded FRP composites are fabricated by pulling continuous fibers through a resin bath and then through a shaping die. This process created continuous profiles with consistent cross-sectional shapes. Pultruded FRP is used in structural applications, such as beams, tubes, and rods [137].
2. Filament-wound process: Filament-wound FRP composites are created by winding continuous fibers, typically glass or carbon, around a rotating mandrel and impregnating them with resin. This method produces cylindrical or tubular shapes and is commonly used in pressure vessels and pipes [138].
3. Hand lay-up process: Hand lay-up FRP involves manually applying layers of fiber and resin to a mold. This is a versatile method for creating custom FRP products or for small-area repair of FRP products. However, they may exhibit variations in their quality and consistency [139].
4. Spray-up process: Spray-up FRP involves spraying a mixture of chopped fibers and resin onto a mold. This is a fast and cost-effective method to obtain large and simple shapes [140].

**Figure 2:**
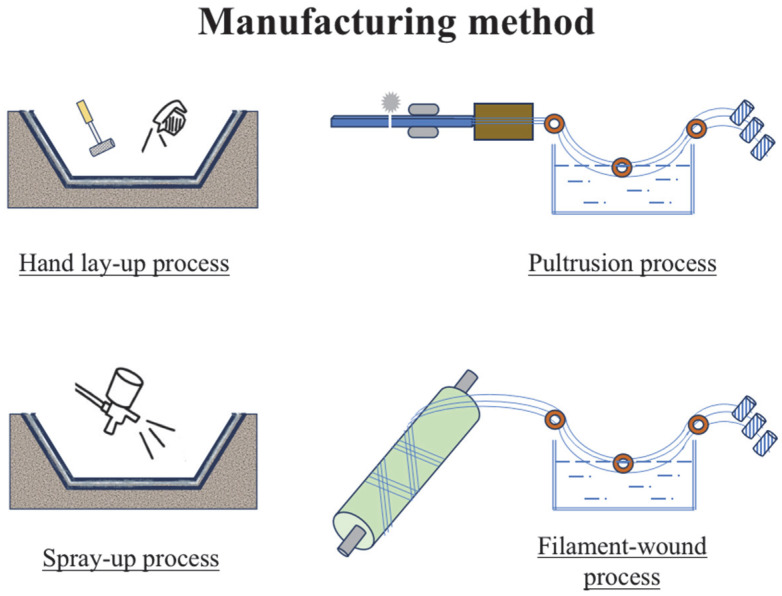
Schematic of FRP manufacturing methods.

Filament wounds or filament windings are typically used for large-diameter pipes and can be efficiently produced through continuous winding. However, pultrusion is more suitable for smaller and singular designs such as tubes, L-channels, and C-channels. The efficiency of the hand lay-up process is limited because it is purely handmade, whereas the spray-up process has the potential to increase productivity. Figure 2 shows the schematic of the aforementioned processes.

#### 3.2.1. Vacuum- and Pressure-Assisted Resin Infusion Methods

Vacuum-assisted resin infusion (VARI) or vacuum-assisted resin transfer molding (VARTM) is used to produce large-scale composite structures, including FRP pipes. This process involves placing dry fiber reinforcements into a mold and using vacuum pressure to infuse the resin through the fiber network, ensuring complete wetting and minimizing void content [141,142,143].

The process begins by laying dry fibers into a mold, which is then sealed in a vacuum bag. A vacuum pump was used to evacuate the air from the mold and create a pressure differential. This pressure difference draws the liquid resin into the mold through the inlet ports, allowing it to saturate the fiber layers under controlled pressure [144]. Once the resin has fully infiltrated the fibers and the curing process is complete, the part is demolded, resulting in a high-quality, void-free composite structure [145,146].

This method has several advantages. This enables a high fiber-to-resin ratio, which significantly improves the mechanical properties of the composite. Compared with hand lay-up and other open-mold methods, VARI/VARTM results in reduced void content. This leads to an enhanced structural integrity and performance of the final product [147]. Additionally, this technique is associated with lower labor costs and is highly scalable for the production of large or complex parts [148,149]. From an environmental perspective, it minimizes resin waste and reduces operator exposure to volatile organic compounds (VOCs).

However, there are some limitations to the study. The process requires specialized tooling and equipment, which may involve significant initial investments. Process control is critical to ensure complete impregnation of the fibers and avoid issues such as resin starvation. Currently, this method is primarily limited to thermosetting resins, although research and development are ongoing to extend this technique to thermoplastic resins.

In FRP pipe manufacturing, vacuum- and pressure-assisted resin infusion methods are particularly valuable. They are commonly used for the production of large-diameter FRP pipes, pipe fittings, and custom pipe sections, which require high mechanical performance and dimensional stability. Industries such as marine, chemical, and oil and gas industries benefit significantly from this technology [150,151]. These sectors require materials with excellent corrosion resistance and mechanical strength, both of which are characteristics of FRP pipes manufactured using VARI/VARTM. By incorporating vacuum- or pressure-assisted resin infusion methods, manufacturers can achieve superior structural performance and quality control, making this technique increasingly popular for use in high-specification FRP piping systems [152].

By incorporating vacuum- or pressure-assisted resin infusion methods, manufacturers can achieve superior structural performance and quality control, making this technique increasingly popular for high-specification FRP piping systems.

**Figure 3:**
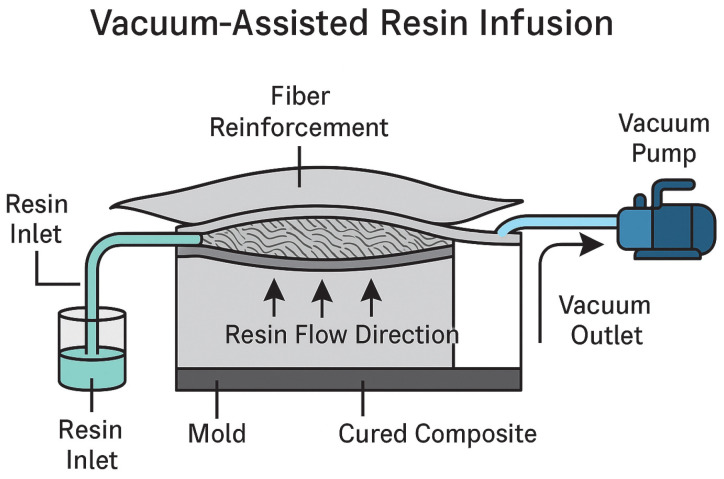
Schematic representation of vacuum-assisted resin infusion process for FRP manufacturing.

Figure 3 illustrates the schematic layout of the VARI process, highlighting the flow of the resin from the inlet reservoir to the fiber reinforcement under vacuum pressure. The use of a sealed vacuum bag and controlled resin flow ensured the complete impregnation of the fiber layers, resulting in a high-quality, void-free composite. This process not only optimizes the mechanical performance, but also reduces material waste, making it an attractive option for scalable and sustainable FRP pipe production [152].

#### 3.2.2. Materials and Manufacturing Technologies for FRP Pipe Production

FRP pipes typically consist of a composite laminate structure composed of various materials, including reinforcement fibers, matrix resins, fillers, and additives. Reinforcement fibers play a crucial role in the strength and stiffness of composites. Glass fibers, such as E-glass and S-glass, are the most common owing to their cost-effectiveness, corrosion resistance, and high tensile strength [153]. Carbon fibers are used for high-pressure or high-stiffness applications, offering superior strength-to-weight ratios. Aramid fibers such as Kevlar are applied in impact-resistant or ballistic protection scenarios [154].

Matrix resins serve as binding agents in the composites. Thermosetting resins, including polyester, vinyl ester, and epoxy resins, such as GRE, GRVE, and GRUP, are widely used because of their excellent mechanical and thermal properties after curing. Thermoplastic resins such as polypropylene, PEEK, and PET are gaining attention for their recyclability, improved impact resistance, and thermoformability [155].

Fillers and additives, such as silica, alumina trihydrate, and fire retardants, may be incorporated to enhance the thermal, chemical, and flame resistance of the composite.

Several manufacturing technologies have been employed in the production of FRP pipes, each offering different performance characteristics and cost implications [156]. Filament winding involves winding continuous fibers under tension over a rotating mandrel in precise patterns such as hoop, helical, or polar. This method is suitable for high-pressure pipes with consistent fiber alignment and can be automated for large-scale production. Centrifugal casting, or spinning, involves placing resin and fibers in a rotating mold to create a dense, smooth inner surface. This method is commonly used for sewage treatment, drainage, and low-pressure applications. Pultrusion involves pulling continuous fibers through a resin bath and heated die to produce constant cross-sectional profiles, such as pipe supports or structural members. Hand lay-up and spray-up involve manually applying fibers and resin to a mold or form, making them suitable for custom or low-volume production, although they offer lower consistency than automated methods. VARI/VARTM provides high-quality void-free laminates for large-diameter or high-specification pipes. Fusion bonding for thermoplastic pipes involves heat-fusing thermoplastic layers to form chemically resistant, recyclable piping systems.

These diverse materials and manufacturing technologies allow the production of FRP pipes that are tailored to meet specific performance requirements and application demands [157].

### 3.3. Common Defects in FRP Materials During Manufacturing

Although FRP materials offer several advantages, they are not immune to defects during manufacturing. Identifying and addressing defects is crucial for ensuring product integrity. Common defects include the following:

1. Voids: Voids are areas within composite materials that contain air pockets that weaken their structure and reduce their strength [158]. The formation of voids can be attributed to several factors including incomplete resin wetting, entrapped air bubbles during lay-up or infusion, and resin shrinkage. To prevent the formation of voids, it is crucial to ensure thorough mixing of the resin and proper wetting of the fibers [159]. Additionally, vacuum- or pressure-assisted resin infusion should be implemented to reduce air entrapment, and the curing conditions should be controlled to minimize resin shrinkage [160].
2. Delamination: Delamination is a phenomenon that occurs when the layers of reinforcement fibers separate, leading to a reduction in the material’s integrity and expansion during service while increasing the stress and impact [161]. The formation of delamination can be attributed to several factors, including poor bonding, inadequate pressure during curing, and repeated thermal stress. To prevent delamination, it is crucial to ensure that adequate pressure is applied during curing and good surface preparation. Additionally, the design components should be minimized to reduce the thermal cycling stress and prevent delamination [162,163,164].
3. Fiber misalignment: Improper fiber alignment can result in the creation of weak points or anisotropy in the composite material. This may be caused by inadequate equipment maintenance, incorrect positioning during the lay-up process, or inadequate training of personnel. Regular equipment maintenance and personnel training are essential for preventing fiber misalignment [165,166].
4. Resin-rich or resin-poor areas: An inconsistent distribution of resin or uneven fiber distribution can result in areas with either excessive or inadequate resin content, which can negatively impact the structural integrity [158]. This is caused by improper resin application during the lay-up process, and the resin flow within the mold may not be consistent. To prevent the need for careful control of resin application, it is vital to ensure even coverage and consistent resin flow.
5. Surface imperfections: The presence of cracks, bubbles, or other irregularities on the surfaces of FRP components can negatively affect their aesthetic appearance and functionality. The appearance of these imperfections can be attributed to several factors, including defects or damage to the mold, contaminants, or foreign particles present during the lay-up process, and variations in the curing process. To prevent the formation of surface imperfections, it is recommended to regularly inspect and maintain the molds, ensure a clean and controlled environment during the lay-up process, and monitor and control curing conditions to ensure consistency.

Gaining insight into the underlying causes of these defects is of paramount importance to proactively address them during the manufacturing process. It is worth noting that additional defects can manifest when FRP materials are in service, depending on the particular environment and service, such as joint defects, matrix cracks, and fiber breaks.

#### Geometric Deformation Defects

In addition to internal defects, such as voids, porosity, and delamination, geometric deformation defects pose significant challenges in the manufacture of FRP pipes and composite components. These defects include spring-in, warpage, and shrinkage, which can compromise the dimensional accuracy, fit-up, and long-term mechanical performance of the final product, respectively.

1. Spring-in: Spring-in manifests as an angular deviation in the curved or angled parts when they relax after demolding. This defect is commonly observed in elbows, flanges, and pipe fittings, where the final angle exceeds the intended dimensions, leading to assembly and alignment complications. The root causes of spring-in include residual stresses within the composite, differential thermal contraction between the fiber and resin matrix, and uneven curing shrinkage. To mitigate spring-in, manufacturers can optimize tooling design and cure schedules, implement symmetric fiber layups to balance stresses, and utilize process simulations to predict and compensate for the expected deformations. Additionally, post-curing treatments or mechanical trimming can be employed to restore dimensional tolerances [167].
2. Warpage: Warpage occurs when a component is distorted out of the plane, resulting in dimensional instability or surface waviness. In FRP pipes, warpage can affect the roundness, straightness, and flange flatness of the product, thereby complicating sealing and joint integrity. The factors contributing to warpage include asymmetric layups, uneven curing, thermal gradients, and tooling design limitations [168]. To prevent warpage, it is essential to design symmetric fiber layups, control curing conditions to ensure uniformity, and optimize tooling to minimize the thermal gradients. Advanced process simulations can also be used to predict and compensate for warpage tendencies, while post-processing steps, such as controlled cooling and mechanical straightening, can help restore dimensional accuracy [169,170].
3. Shrinkage: Shrinkage is another critical geometric deformation defect that occurs during the curing or cooling of FRP components. As the resin cures and cools, it contracts, potentially leading to dimensional reductions in pipe diameter or thickness [171]. Excessive shrinkage can cause fitment issues and compromise the pressure containment capability of the pipes. The primary causes of shrinkage include the inherent volumetric contraction of the resin during curing and the differential cooling rates between the composite and mold [172,173]. To mitigate shrinkage, manufacturers can select low-shrinkage resin formulations, optimize curing cycles to control cooling rates, and employ shrinkage-compensating tooling designs. Additionally, incorporating reinforcing fibers with appropriate coefficients of thermal expansion can help reduce shrinkage-related deformation [174,175].

Recognizing and mitigating these geometric deformation defects is essential to ensure that FRP piping systems satisfy stringent dimensional and performance requirements, particularly in critical infrastructure and industrial applications.

## 4. Using Digital Technologies to Enhance NDT Performance

In the rapidly evolving world of technology, NDT has transitioned from traditional inspection methods to more sophisticated techniques that harness various technologies to achieve faster and more accurate detection. A comprehensive literature review shows that these technologies can be broadly categorized into numerical modeling, simulation, ML, and DL. Some researchers have combined these approaches to enhance NDT performance.

This section aims to showcase specific success case studies that highlight the application of numerical modeling, simulation, ML, DL, or a combination of these approaches in various NDT methods for composites or other materials. The practical benefits and advantages of these digital technologies in the field of NDT can be obtained by examining these success stories.

### 4.1. Numerical Modeling and Simulation: Purpose and Classification of Methods

As the complexity of FRP materials and their structural applications continues to grow, the limitations of traditional NDT methods, such as operator dependency, limited depth resolution, and insufficient sensitivity to composite-specific defects, have become increasingly apparent. To address these challenges, numerical modeling and simulation have emerged as essential tools for enhancing NDT performance by providing predictive insights, optimizing inspection parameters, and reducing reliance on costly experimental trials.

The purpose of this section is threefold.

1. To categorize the leading numerical methods applied in NDT, including FEM, BEM, finite integration technique (FIT), Monte Carlo simulation, and semi-analytical models.
2. To demonstrate their practical applications across various NDT techniques, they were supported by findings from recent literature.
3. To summarize their comparative advantages, limitations, and reported performance metrics to assist practitioners and researchers in selecting appropriate methods for specific NDT scenarios.

The following subsections group the literature by modeling methodology and highlight their impact on the advancement of NDT capabilities for composite materials, particularly FRP structures.

#### 4.1.1. Finite Element Method Applications in NDT

The FEM is among the most widely applied numerical modeling techniques in advanced NDT research. Its ability to handle complex geometries, multiphysics interactions, and material anisotropy makes it particularly suited for studying wave propagation, stress analysis, and thermal behavior in FRP materials [176,177,178].

The FEM discretizes a complex structure into smaller elements, allowing researchers to simulate how ultrasonic waves, thermal energy, or mechanical stresses interact with defects such as delamination, voids, or cracks. Multiple studies have successfully employed the FEM to enhance the predictive capability and reliability of various NDT techniques.

- Acharjee and Bandyopadhyay [179] applied FEM to assess the structural integrity of fire-damaged reinforced concrete members, demonstrating how temperature load impacts structural responses and validating their computational predictions with experimental results.
- Munalli et al. [180] combined FIT and FEM to simulate microwave-based NDT to detect damage in CFRP materials. Their study analyzed scattering parameters (S-parameters) to differentiate between healthy and damaged regions, showing a good correlation with experimental data.
- Evans et al. [181] integrated the FEM with experimental validation to predict the type, location, and extent of impact damage in CFRP laminates subjected to low-velocity impacts. Their hybrid modeling-experimental approach provided a comprehensive understanding of the damage mechanisms.
- Feito et al. [182] used FEM to simulate the mechanical behavior of open-hole CFRP laminates under tensile and fatigue loading and achieved close agreement with the experimental strain distribution and crack progression results.
- Fang and Maldague [40] utilized the FEM to model the thermal response of CFRP specimens with controlled defects. Their simulation data were subsequently used to train a gated recurrent unit (GRU) deep learning model, which successfully quantified the defect depth from thermal signals.
- Ratsakou et al. [183] validated a semi-analytical truncated region eigenfunction expansion (TREE) model by comparing it with FEM-based COMSOL simulations for the IRT of delaminated planar structures, confirming FEM’s role as a validation benchmark.
- Notebaert et al. [184] demonstrated the use of COMSOL Multiphysics FEM simulations to model the active thermographic inspections of additively manufactured composite parts, achieving a high degree of agreement between the simulated and experimental results.
- Kim et al. [185] focused on modeling and simulating IRT to detect subsurface defects in hydroelectric penstocks. The study used ANSYS version 19.2.0 to build a 3D penstock model and simulate lock-in infrared thermography.

These studies illustrate the versatility and robustness of the FEM in simulating a wide range of NDT scenarios, from ultrasonic wave propagation to thermal and mechanical analyses. Despite its computational demands, the FEM remains a powerful tool for predicting defect responses, optimizing inspection parameters, and enhancing the reliability of NDT for FRP materials.

#### 4.1.2. Boundary Element Method (BEM) and Semi-Analytical Models

Although FEM is well established for simulating multi-physics and complex geometries, its computational cost can be prohibitive, particularly for large-scale or frequency-domain simulations. The BEM and semi-analytical models offer computationally efficient alternatives for specific NDT applications, particularly in electromagnetic and wave-based inspections [186,187].

BEM focuses on discretizing only the boundary of the geometry rather than the entire volume, significantly reducing the computational resources for problems involving infinite or semi-infinite domains, such as electromagnetic field simulations. The BEM has shown a particular value in ECT and POD modeling [188].

- Baskaran et al. [189] developed a BEM-based framework to model the eddy current responses for flaw detection in conductive materials. Their study integrated BEM results with Gaussian statistical models to improve POD estimation, demonstrating that BEM could accurately compute impedance changes with less than 5% error compared to the experimental data.
- Apostol et al. [72] proposed analytical and numerical models based on Maxwell’s equations to enhance ECT signal interpretation. Their approach validated the numerical results using semi-analytical solutions, reinforcing the reliability of the BEM in electromagnetic NDT.
- Hachi et al. [190] employed a hybrid 3D FEM and magnetic vector potential formulation to simulate the eddy current density distribution in CFRP composites. Their work highlighted the anisotropic electrical behavior of CFRP, demonstrating BEM’s applicability in complex material characterizations.

Semi-analytical models, such as eigenfunction expansions and heat diffusion equations, provide rapid approximate solutions for thermal, ultrasonic, and electromagnetic phenomena [191]. Although less flexible than numerical methods for complex geometries, they offer significant computational speed for specific controlled scenarios.

- Ratsakou et al. [183] developed a TREE semi-analytical model to simulate heat propagation in delaminated structures. Their results showed strong agreement with the FEM-based COMSOL simulations, validating the efficiency and accuracy of their approach for infrared thermography applications.
- Apostol et al. [72] further demonstrated the usefulness of analytical modeling in eddy current analysis, providing closed-form solutions for magnetic vector potentials in layered media.
- Hachi et al. [190] validated their numerical results using analytical solutions for unidirectional CFRP plates, ensuring the consistency and reliability of their computational models.

Although BEM and semi-analytical methods may not match FEM’s versatility for highly detailed or nonlinear problems, they offer computational advantages for specialized applications, particularly when combined with POD modeling or signal interpretation frameworks. Their validation through comparison with FEM or experimental data enhanced the confidence in their predictive capabilities.

#### 4.1.3. Finite Integration Technique (FIT), Monte Carlo Methods, and Other Numerical Approaches

##### Finite Integration Technique

FIT has gained traction as a computational method particularly suited for high-frequency electromagnetic simulations such as microwave and millimeter-wave NDT. FIT discretizes Maxwell’s equations in integral form, making it well-suited for analyzing the interaction of electromagnetic waves with complex materials.

- Munalli et al. [180] combined FIT and FEM to simulate the use of microwave NDT techniques for damage detection in CFRP. Their study demonstrated that variations in S-parameters could effectively distinguish between healthy and defective areas. FIT simulations correlated well with experimental measurements, confirming their value for electromagnetic wave-based NDT applications.

##### Monte Carlo Simulation Methods

Monte Carlo methods are statistical simulation techniques that model the probabilistic behavior of complex systems through repeated random sampling [192,193]. These methods are particularly valuable in radiographic NDT, where they simulate X-ray and gamma-ray interactions with materials to optimize imaging parameters and reduce safety risks [194].

- Mousa et al. [195] used the GEANT4 GATE toolkit to simulate computed radiography testing (CRT) of carbon steel plates and pipes, demonstrating improved image quality and system optimization without the need for extensive physical trials.
- Sari et al. [196] applied Monte Carlo simulations to model gamma-ray backscatter for detecting voids in concrete, validating the method using experimental data to ensure accurate calibration.
- Kumar et al. [197] optimized the radiographic parameters for inspecting nuclear fuel reprocessing tanks using aRTist, a radiography simulation software, and achieved good alignment with experimental observations.

##### Other Numerical Techniques and Hybrid Approaches

Several other specialized numerical tools and hybrid approaches have also been reported in the literature, expanding the range of simulation techniques applicable to advanced NDT.

- Osipov et al. [198] introduced a high-performance algorithm for simulating digital radiography testing (DRT) of large, complex industrial components, enabling realistic image generation without extensive experimental testing.
- Rodat et al. [199] presented a metamodeling simulator that integrates human factors into MAPOD studies, allowing a realistic simulation of human-influenced inspection outcomes.
- Lei et al. [200] used SimSUNDT software for a fully simulation-based POD study of PAUT, demonstrating the feasibility of generating reliable POD curves without extensive physical experiments.

These alternative methods highlight the breadth of simulation capabilities available to researchers and practitioners in NDT. Although each technique has its specific strengths and limitations, their combined use in hybrid modeling frameworks often yields more robust, cost-effective, and accurate NDT solutions, as summarized in Table 4.

#### 4.1.4. Analytical Insights and Selection Considerations

The reviewed studies collectively demonstrate that no single modeling method is universally superior. The selection of a suitable numerical approach depends on the following:

- Type of NDT technique being modeled (ultrasonic, radiographic, electromagnetic, thermal, etc.).
- Complexity of component geometry (simple plate versus complex 3D structure).
- Nature of materials (isotropic metals vs. anisotropic composites).
- Computational resources and time constraints.
- Required output accuracy and validation requirements.
- FEM is the most versatile and widely validated method, particularly for multiphysics problems in complex composite structures.
- The BEM and semi-analytical models offer faster computations for specific electromagnetic and thermal applications, making them ideal for iterative design studies or POD evaluations.
- Monte Carlo methods are used in radiography optimization to reduce experimental exposure requirements and radiation safety risks.
- Hybrid and meta-modeling approaches provide a realistic simulation of human and environmental variability, making them valuable for risk-based decision-making frameworks, such as MAPOD.

This comparative analysis supports the strategic integration of multiple simulation methods to complement each other, thereby enhancing the reliability, efficiency, and cost-effectiveness of advanced NDT solutions for fiber-reinforced polymer materials and piping systems.

### 4.2. Machine Learning and Deep Learning in NDT

The rapid advancement of ML and DL has introduced transformative capabilities in NDT, particularly in automating defect detection, improving classification accuracy, and reducing operator dependence. Unlike conventional signal processing or rule-based approaches, ML and DL techniques learn from data patterns, enabling them to recognize complex defect signatures in various NDT modalities, including ultrasonic, radiographic, thermographic, and electromagnetic inspection.

#### 4.2.1. Overview of Machine Learning Models for NDT

Recent studies have explored multiple ML algorithms, each offering unique advantages and tradeoffs. As summarized in Table 5, the commonly used models include the following:

- Support vector machines (SVM): Owing to their high classification accuracy, SVMs have been effectively applied in defect classification tasks such as identifying crack-like and pore-like defects in ultrasonic phased array images. The Poly-SVM variant demonstrated classification accuracies of up to 93%, outperforming several competing models [201,202,203].
- Decision trees (CART): The CART models offer high interpretability, allowing inspectors to trace the decision-making process. However, their performance may be lower than that of other models on complex datasets, owing to overfitting risks.
- K-nearest neighbors (KNN): The KNN is a simple yet powerful algorithm that classifies defects based on proximity in the feature space. Its performance depends on the choice of k and the distance metric, and it can be computationally demanding for large datasets [204,205].
- Naïve Bayes: As a probabilistic classifier, Naïve Bayes is valued for its speed and efficiency but assumes independence among features, which may not always hold in complex inspection scenarios [206,207].

These models have been applied in various contexts, including weld defect detection in radiographic images [208], impact damage classification in CFRP structures using thermography, and multisensor data fusion for defect characterization [209].

Successful deployment of ML models in NDT requires the availability of high-quality, accurately labeled datasets that represent a balanced distribution of defect and non-defect instances. Before model training, the collected data must undergo essential preprocessing procedures, including normalization to standardize data ranges, noise reduction to improve signal clarity, and feature extraction to convert raw sensor data into structured input suitable for ML algorithms [210]. To ensure model robustness and generalization to unseen data, the dataset should be systematically partitioned into separate training, validation, and testing sets. Finally, the trained models must be evaluated using established performance metrics such as accuracy, precision, recall, and F1-score to verify their reliability and effectiveness in practical NDT applications [209].

#### 4.2.2. Overview of Deep Learning in NDT

DL, a subfield of ML and artificial intelligence (AI), has emerged as a transformative approach to NDT [211,212]. Unlike traditional numerical modeling or rule-based analysis, DL leverages representation learning and numerical optimization to automatically learn complex patterns from data, without requiring predefined features or handcrafted rules.

DL models, particularly deep neural networks (DNNs), consist of multiple hidden layers that hierarchically extract features from the raw data. This architecture enables them to handle high-dimensional inputs, such as images, waveforms, and sensor signals. DL methods have demonstrated remarkable performances in classification, segmentation, and regression tasks across various NDT modalities [213,214]. The common software tools and system configurations used for the DL are listed in Table 6.

Recent studies have demonstrated the effectiveness of DL in enabling automated defect detection, segmentation, and classification using various NDT techniques. For example, Hena et al. [215] applied a U-Net segmentation model to DRT images, specifically investigating how the signal-to-noise ratio (SNR) and contrast-to-noise ratio (CNR) influence the defect recognition performance. Fotouhi et al. [216] explored autonomous damage classification in composite materials using several DL architectures, including AlexNet, ResNet-50, and custom convolutional neural networks (CNNs) [217]. Among these, AlexNet achieved the highest accuracy, ranging from 87% to 96%, in identifying damage types and severities.

Wei et al. [218] contributed by developing the PVC-Infrared dataset for pulsed thermography applications and evaluating the performance of DL-based instance segmentation models on this dataset. Their findings showed that appropriate data reduction techniques could minimize the dataset size without compromising segmentation accuracy. In the UT domain, Virkkunen et al. [219] demonstrated that incorporating data augmentation through virtual flaw generation significantly improved DL model performance, allowing it to achieve human-level accuracy in flaw detection.

Trouvé-Peloux et al. [220] applied semantic segmentation neural networks to UT C-scan data and showed that these DL methods outperformed traditional unsupervised machine learning and classical analysis techniques in accurately localizing defects in composite materials. Similarly, Yosifov et al. [221] compared traditional segmentation algorithms, such as k-means clustering, watershed, and Otsu thresholding, with DL-based models, such as U-Net and V-Net, on XCT data. Their study demonstrated that DL methods achieved superior defect detection limits and improved the POD curve estimation.

In the area of thermographic analysis, Wei et al. [222] developed DL models for detecting impact damage in curved CFRP laminates using mid-wave and long-wave infrared thermography, achieving F1-scores of 92.7% and 87.4%, respectively. Guo et al. [223] applied the InceptionTime DL architecture to AET data, achieving approximately 99% classification accuracy in identifying various damage modes, including fiber breakage, matrix cracking, and delamination. Finally, Du et al. [224] advanced casting defect detection in X-ray images by integrating feature pyramid networks.

Effective deployment of DL models in NDT depends on the availability of large, diverse datasets that are accurately annotated to reflect various defect types and conditions. To improve model generalization and address data limitations, data augmentation techniques such as geometric transformations or noise injection are commonly applied to expand the training dataset. During the model development phase, strategies such as dropout regularization, early stopping, and cross-validation are essential to prevent overfitting and ensure the model performs reliably on unseen data [221]. Given the computational intensity of training DNNs, access to specialized hardware resources such as GPUs or cloud-based platforms is often necessary to achieve practical training times and scalable deployment in real-world inspection scenarios [225].

#### 4.2.3. Practical Integration of ML and DL into NDT Workflows

Although these models demonstrate strong technical capabilities, their practical value is maximized when integrated into complete NDT workflows. A typical digital-physical NDT process involves the following stages.

1. Virtual inspection planning using simulation
  Numerical methods such as FEM, BEM, or FIT are used to simulate defect responses and optimize inspection parameters before physical testing.
2. Physical Data Acquisition
  Standard NDT techniques, such as PAUT, IRT, XCT, and ECT, are employed to collect raw inspection data, including ultrasonic waveforms, thermal images, and radiographic projections.
3. Data Preprocessing and Feature Extraction
  Raw signals or images are pre-processed to extract relevant features such as amplitude, frequency content, temperature gradients, or geometric descriptors.
4. Machine Learning-Based Defect Classification
  The pre-processed features are fed into ML models such as SVM, KNN, CART, or Naïve Bayes to automatically classify the type, location, and severity of detected defects. This step significantly reduces human interpretation efforts and standardizes defect assessments.
5. Model Validation and Performance Tuning
  ML models are trained and validated using historical datasets or synthetic data generated from simulations to ensure robustness and generalizability to real-world inspections.
6. Integration with digital twin and predictive maintenance platforms
  Advanced workflows may further integrate ML outputs with digital twin models or IoT sensor networks, thereby enabling real-time monitoring, predictive maintenance, and lifecycle management.

#### 4.2.4. Deployment Considerations, Data Management, and Industrial Integration

Although ML and DL have shown promising results in controlled studies, achieving practical and scalable industrial deployment requires addressing several operational and data management challenges. Real-time processing capability is a critical requirement, particularly for applications where inspection speed and decision-making are time-sensitive. Optimizing model inference speed and computational efficiency ensures that ML and DL models can be deployed on embedded systems, edge devices, or integrated directly into NDT equipment without causing operational delays.

Seamless integration into existing inspection workflows requires compatibility with various data acquisition systems, NDT software platforms, and industrial digital twin environments. Models must be packaged in a way that allows them to interact with live sensor data streams, enabling automated defect detection and real-time decision support. Furthermore, establishing closed-loop feedback mechanisms is essential to continuously update models with new inspection data, improving model robustness and adapting to changes in material types, defect characteristics, and operating conditions.

Data management also plays a crucial role in ensuring the reliability and sustainability of ML and DL-based NDT solutions. Organizations must implement secure and structured data storage solutions that facilitate easy retrieval and traceability. Metadata documentation, such as sensor configuration, inspection conditions, and labeling criteria should accompany datasets to ensure reproducibility and auditability. Compliance with data privacy and industry-specific regulations is also essential when handling sensitive or proprietary inspection data.

To fully leverage the potential of ML and DL in predictive maintenance and lifecycle management, these models should be integrated with broader asset management systems, including digital twins and IoT-based monitoring platforms. By doing so, organizations can transition from reactive maintenance practices to proactive and predictive strategies, reducing downtime, improving safety, and optimizing asset performance over their entire lifecycle.

#### 4.2.5. Advantages and Challenges of ML and DL Integration in NDT

The reviewed studies collectively demonstrate that ML and DL techniques offer significant potential for transforming NDT practices across a wide range of materials, defect types, and inspection modalities. Both ML and DL contribute to automating complex data interpretation, significantly reducing inspection time and operator dependency. DL techniques, in particular, achieve high classification and segmentation accuracy, with reported performances reaching up to 99% in identifying defects, such as cracks, porosity, delamination, and impact damage.

These methods further enable the automation of feature extraction, eliminate the need for manual feature engineering, and enhance detection consistency. Their scalability across various NDT data types, including ultrasonic waveforms, radiographic images, infrared thermograms, and acoustic emission signals, makes them applicable in a wide range of industrial scenarios, including FRP pipes and composite structures.

In addition to improving defect detection performance, ML and DL models support enhanced decision-making through MAPOD studies. These frameworks integrate simulation and experimental data to provide quantitative reliability assessments, ensuring that inspection procedures meet the regulatory and safety requirements.

Moreover, the compatibility of ML and DL with Industry 4.0 frameworks positions these technologies as key enablers of real-time monitoring, predictive maintenance, and digital asset management. By integrating advanced data analytics with IoT sensor networks and digital twin platforms, organizations can shift from reactive to proactive maintenance strategies, thereby improving asset reliability and operational efficiency.

However, these benefits are challenging to achieve. The limited availability of high-quality, labeled training datasets, especially for specialized applications such as FRP pipes, remains a significant barrier to their widespread adoption. In addition, the computational demands of training deep models require access to specialized hardware or cloud-based platforms, which may not be readily available to all organizations. Overcoming these challenges will require further research, industry collaboration, and the development of application-specific datasets and validation protocols.

## 5. Summary and Discussion

The integration of digital technologies into NDT methods has revolutionized the detection of defects in composite materials, enabling faster and more accurate assessments. This literature review highlights significant advancements in NDT performance through the application of numerical modeling, simulation, ML, and DL.

Numerical modeling and simulation techniques, particularly FEM, have been pivotal for enhancing NDT capabilities. The FEM has been extensively employed to analyze the behavior of complex materials under various conditions. For instance, studies demonstrating the effects of thermal loads on fire-damaged reinforced concrete structures illustrate FEM’s effectiveness in predicting structural failure. Similarly, FEM has been used to characterize the mechanical behavior of CFRP laminates under quasi-static tensile and fatigue loading, with findings showing good agreement between the experimental and numerical results. This alignment underscores the reliability of the method for predicting crack initiation and propagation, which is critical for assessing the integrity of composite structures.

Additionally, the use of commercial software such as COMSOL and ANSYS has facilitated advanced numerical modeling. Researchers have effectively simulated the thermal response of CFRP specimens using COMSOL, whereas others have validated comprehensive modeling approaches for additively manufactured composites, indicating the robust applicability of these tools in practical scenarios.

Moreover, the enhancement of RT through numerical modeling has streamlined the design of DRT systems, allowing for efficient system development with minimal experimental testing. The effective simulation of computed radiography techniques using the GEANT4 GATE toolkit further exemplifies how numerical modeling can optimize traditional NDT methods.

Turning to ML, both ML and DL have emerged as transformative tools in the realm of NDT. Various ML algorithms, including NN and XGB, have been successfully used to predict the mechanical properties of composite materials, showing high predictive accuracy, particularly for VIG-type composite panels. The integration of pulsed thermography with ML has further advanced the automated detection and classification of impact damage in CFRP structures, revealing that ML approaches can outperform conventional statistical analysis methods.

The application of advanced image-processing techniques combined with ML methods for weld defect detection has also shown promising results. For instance, the MSVM classifier effectively identifies and classifies a range of weld defects, underscoring the potential of ML to enhance inspection accuracy in critical applications.

DL methodologies have taken these advancements further, particularly for automated defect recognition. Research exploring the use of DL neural networks for DRT images has revealed how image quality parameters such as SNR and CNR significantly influence the performance of models such as U-Net. Additionally, DL approaches have demonstrated high accuracy in classifying the types and severities of composite damage, making them valuable for real-time inspection applications.

### 5.1. Comparative Performance Analysis

To evaluate the effectiveness of the various digital approaches reviewed, Table 7 presents a comparative summary of reported performance outcomes. These include quantitative metrics such as accuracy, POD, F1-scores, and improvement percentages for both modeling- and AI-based techniques.

### 5.2. Key Observations and Industrial Implications

Several key trends emerge from the performance analysis in Table 7.

- Simulation-based techniques (e.g., FEM, BEM, and Monte Carlo) offer reliable pre-inspection planning tools, enabling the optimization of sensor configuration, parameter tuning, and MAPOD evaluation.
- ML methods provide effective solutions for feature-based defect classification, particularly when well-labeled data are available.
- DL approaches are effective in handling large and complex datasets, achieving state-of-the-art performance in classification and segmentation, particularly in imaging-based NDT (e.g., radiography, infrared, and ultrasonic C-scan).
- The combination of simulation and AI methods allows for synergistic workflows such as training ML models on synthetic defect data generated from simulations.
- These approaches are increasingly compatible with Industry 4.0, offering opportunities for real-time monitoring, predictive maintenance, and digital twin integration.

These capabilities are particularly important in FRP pipe systems. Given the anisotropy, multi-layered construction, and curvature of FRP components, traditional NDT methods often struggle with sensitivity and consistency. Advanced digital tools provide the flexibility and accuracy required for early defect detection, automated interpretation, and lifecycle reliability assessment.

Despite these benefits, several challenges remain. These include application-specific datasets, model interpretability, computational resource requirements, and industrial-scale validation. Future work should focus on bridging these gaps by developing open-access defect libraries, refining hybrid simulation-AI frameworks, and deploying field-ready NDT solutions tailored to FRP pipe applications. 

## 6. Conclusions

This comprehensive review has critically examined the state-of-the-art advancements in digital technologies for NDT of FRP pipes, with a particular focus on the roles of numerical simulation, ML, and DL in enhancing defect detection, classification, and system reliability. Section 4 and Section 5 demonstrate that while conventional NDT methods provide foundational capabilities, they fall short when confronted with the inherent complexity, anisotropy, and multiscale defect mechanism characteristics of FRP pipe systems.

The integration of FEM, BEM, and probabilistic modeling has emerged as a paradigm shift, enabling predictive defect behavior analysis, optimization of inspection strategies, and MAPOD assessments with minimized experimental dependency. The application of ML and DL algorithms, ranging from SVM and CNN architectures to advanced semantic segmentation models, has demonstrated remarkable performance in automating defect characterization, reducing human bias, and accelerating data-driven decision-making processes.

However, despite these technological breakthroughs, the review underscores a critical research gap: the limited translation of these digital NDT advancements into field-deployable solutions specifically optimized for FRP piping infrastructure. The unique challenges posed by the layered, anisotropic, and often large-scale nature of FRP pipelines necessitate tailored NDT methodologies that extend beyond generic composite material applications. Furthermore, the scarcity of high-fidelity, domain-specific datasets coupled with the lack of standardized validation frameworks for FRP pipe inspection hinders the widespread adoption of these advanced methods in real-world industrial environments.

To bridge this gap, future research must prioritize the development of integrated digital twin frameworks that combine real-time sensor data, high-resolution simulation models, and AI-driven analytics for the continuous structural health monitoring of FRP pipelines. Collaborative efforts between academia, industry, and standardization bodies are essential for establishing benchmark datasets, validation protocols, and best practices tailored to FRP piping systems. Moreover, advancing explainable AI techniques will be pivotal to ensuring transparency, interpretability, and regulatory acceptance of ML- and DL-based NDT systems.

In summary, although digital and AI-enhanced NDT techniques have demonstrated exceptional potential, their full realization in the context of FRP pipes requires a concerted shift toward application-specific research, validated deployment strategies, and cross-disciplinary collaboration. Such advancements will not only elevate the integrity management of FRP piping assets, but also contribute to the broader digital transformation of the composite materials industry.

## Figures and Tables

**Table 1:** Type of FRP overview.

FRP Composite Type	Reinforcement	Key Properties	Typical Applications	Typical Mechanical Properties
GFRP (Glass Fiber-Reinforced Polymer)	Glass fibers	High strength, corrosion resistance, electrical insulation	Construction, automotive, marine	Tensile Strength: 500–900 MPa, Modulus: 35–55 GPa, Density: 1.8–2.0 g/cm^3^
CFRP (Carbon Fiber-Reinforced Polymer)	Carbon fibers	High strength-to-weight ratio, high stiffness, corrosion resistance	Aerospace, automotive, sporting goods, structural engineering	Tensile Strength: 600–3500 MPa, Modulus: 70–250 GPa, Density: 1.5–1.6 g/cm^3^
AFRP (Aramid Fiber-Reinforced Polymer)	Aramid fibers (e.g., Kevlar)	High tensile strength, impact resistance, flame resistance	Body armor, protective gear, structural applications	Tensile Strength: 3000–3600 MPa, Modulus: 60–125 GPa, Density: 1.44 g/cm^3^
BFRP (Basalt Fiber-Reinforced Polymer)	Basalt fibers	High-temperature resistance	Construction, infrastructure repair and reinforcement	Tensile Strength: 2000–4840 MPa, Modulus: 89–93 GPa, Density: 2.7 g/cm^3^
NFRP (Natural Fiber-Reinforced Polymer)	Natural fibers (e.g., jute, hemp, flax)	Environmental friendliness, low density, flexibility, cost-effectiveness	Automotive interiors, furniture, construction	Tensile Strength: 200–900 MPa, Modulus: 10–30 GPa, Density: 1.2–1.5 g/cm^3^
HFRP (Hybrid Fiber-Reinforced Polymer)	Combination of various fiber types	Balanced properties (strength, stiffness, cost-effectiveness)	Wide range of applications	Varies based on fiber combination

**Table 2:** Summary of resin types, advantages, disadvantages, and typical properties for FRP manufacturing.

Resin Type	Advantages	Disadvantages	Typical Properties
Polyester	Low cost, good chemical resistance, easy to process	Lower mechanical strength, poor elongation	Tensile Strength: 40–100 MPa, Tg: 70–80 °C, Low viscosity
Epoxy	High strength, excellent adhesion, good mechanical properties	Higher cost, sensitive to curing conditions	Tensile Strength: 70–150 MPa, Tg: 120–180 °C, Moderate viscosity
Vinyl Ester	High chemical resistance, good mechanical properties, good toughness	More expensive than polyester, handling precautions required	Tensile Strength: 80–130 MPa, Tg: 90–140 °C, Moderate viscosity

**Table 3:** Summary of common thermoplastic resins used in FRP manufacturing: key properties, advantages, limitations, and applications.

Thermoplastic Resin	Key Properties	Advantages	Limitations	Example Applications
Polypropylene (PP)	Low density, good chemical resistance	Lightweight, cost-effective, corrosion-resistant	Low thermal stability, limited mechanical strength	Chemical pipelines, water systems
Polyethylene Terephthalate (PET)	Good mechanical strength, chemical resistance, recyclable	High impact resistance, good environmental profile	Moisture sensitivity, thermal limitations	Water treatment, automotive parts
Polyetheretherketone (PEEK)	High mechanical and thermal performance	Excellent chemical and thermal resistance, high strength-to-weight	High cost, requires high processing temperature	Aerospace, oil and gas piping
Polyamide (Nylon)	High toughness, moderate moisture absorption	Good impact resistance, flexible processing	Moisture sensitivity, lower chemical resistance than PEEK	Automotive, industrial piping

**Table 4:** Comparative summary of numerical modeling methods.

Modeling Method	Strengths	Limitations	Typical Applications	Reported Performance
Finite Element Method (FEM)	Handles complex geometries, multi-physics, anisotropic materials	High computational cost, especially for 3D or high-frequency simulations	Thermal, ultrasonic, mechanical simulations in CFRP and composites	Up to 95% agreement with experimental results [40,182]
Boundary Element Method (BEM)	Efficient for open-boundary electromagnetic problems	Limited to linear, simpler geometries	Eddy current testing (ECT), POD modeling	<5% impedance error compared to experiments [189]
Finite Integration Technique (FIT)	Suitable for high-frequency electromagnetic wave simulation	Limited multi-physics capabilities	Microwave and millimeter-wave NDT in CFRP	High correlation in S-parameter analysis [180]
Monte Carlo Methods	Accurate stochastic modeling for radiation transport and scattering	Computationally intensive, requires validation	Radiography testing (DRT, CRT), gamma backscatter	Improved image quality, validated by experiments [195,196,197]
Semi-Analytical Models	Rapid computation for specific scenarios, validation benchmarking	Limited flexibility for complex geometries	Infrared thermography, Eddy current analysis	<10% deviation from FEM/computational models [72,183]
Hybrid and Meta-Modeling Approaches	Integrates simulation, human factors, and data-driven analysis	Method-specific limitations	MAPOD, human-influenced inspection simulation	Effective simulation of human variability and POD [199,200]

**Table 5:** Summary of four machine learning models applied for defect classification in NDT imaging.

ML Model	Description
Support Vector Machine (SVM)	-Supervised learning algorithm for classification and regression. -Identifies the optimal hyperplane that maximizes class separation margin. -In this study, a polynomial kernel SVM (Poly-SVM) achieved the highest accuracy (93%) in classifying crack-like and pore-like defects in TFM images.
CART Decision Tree	-A tree-based supervised learning algorithm that partitions data based on feature splits. -Produces easily interpretable classification paths. -May underperform on complex datasets compared to advanced algorithms.
K-Nearest Neighbors (KNN)	-Non-parametric algorithm that classifies based on the nearest neighbors in feature space. -Highly dependent on distance metrics (e.g., Euclidean) and the choice of k-value. -Computationally intensive on large datasets.
Naive Bayes	-Probabilistic classifier based on Bayes’ Theorem. -Assumes feature independence, which may not hold true in real applications. -Offers fast training but can lack accuracy on complex or correlated data.

**Table 6:** Deep learning software frameworks and hardware platforms.

Software Frameworks	Hardware Platforms
TensorFlow: An open-source machine learning library developed by Google is widely used to build and deploy deep learning models.	Graphics processing units (GPUs): Deep learning models are computationally intensive, and GPUs with parallel processing capabilities are widely used to accelerate the training and inference of deep neural networks.
PyTorch: An open-source machine-learning library developed by Facebook’s AI Research Lab is known for its flexibility and ease of use.	CPU-based systems: For small-scale or less computationally demanding applications, deep learning models can also be deployed on regular CPU-based systems, especially for inference.
Keras: A high-level neural network API that runs on top of TensorFlow, providing a user-friendly interface for building deep-learning models.	Cloud-based services: Major cloud providers (e.g., AWS, Google Cloud, Microsoft Azure) offer GPU-accelerated virtual machines and managed services for training and deploying deep-learning models.
Caffe/Caffe2: Open-source deep learning frameworks often used for computer vision applications.	Edge devices: Deep learning inference can be performed on edge devices, such as embedded systems, mobile phones, and IoT devices, leveraging specialized hardware, such as tensor processing units (TPUs) or edge AI chips.
MXNet: A flexible and efficient library for deep learning that supports multiple programming languages.	

**Table 7:** Summary of reported accuracy and performance improvements in advanced NDT techniques.

NDT Technique	Digital Enhancement Method	Reported Performance Metric	Reference
Phased Array Ultrasonic Testing (PAUT)	FEM-based simulation and MAPOD	90% probability of detecting defects ≥1 mm at 95% confidence	[200]
Microwave NDT (MWNDT)	FIT and FEM simulation	Accurate S-parameter variation correlated with damage states in CFRP	[180]
Infrared Thermography (IRT)	FEM and GRU Deep Learning	>90% defect depth quantification accuracy	[40]
Eddy Current Testing (ECT)	BEM with Gaussian modeling	Impedance prediction with <5% error	[189]
Radiography Testing (DRT/CRT)	Monte Carlo simulation (GATE Toolkit)	Improved image quality and 30% reduction in exposure time	[195,196]
Ultrasonic TFM Imaging	Poly-SVM ML classification	93% accuracy in classifying crack-like and pore-like defects	[226]
Thermographic Damage Detection	Cube SVM ML classification	78.7–93.5% accuracy in CFRP damage detection	[210]
Radiographic Weld Inspection	MSVM ML classification	High precision in multi-class weld defect detection	[208]
Composite Damage Detection	CNN (AlexNet) DL classification	87–96% accuracy in damage type and severity classification	[216]
Pulsed Thermography	DL instance segmentation	Data reduction with maintained segmentation performance	[218]
Ultrasonic Flaw Detection	DL with virtual flaw data augmentation	Human-level performance in flaw detection	[219]
UT C-scan Defect Localization	Semantic segmentation DL models	Superior defect localization compared to traditional ML	[220]
XCT Image Analysis	U-Net and V-Net DL segmentation	Enhanced POD curve estimation and defect detection limits	[221]
Impact Damage in CFRP	DL models on midwave and longwave IRT data	F1-scores of 92.7% (midwave) and 87.4% (longwave)	[222]
Acoustic Emission Analysis	InceptionTime DL classification	~99% accuracy in fiber breakage, matrix cracking, and delamination detection	[223]
Casting Defect Detection	FPN and RoIAlign DL models	23.6% improvement over Faster R-CNN in small defect localization	[224]

## Data Availability

No new data were created in this study.
